# Functional connectivity alterations in the thalamus among patients with bronchial asthma

**DOI:** 10.3389/fneur.2024.1378362

**Published:** 2024-05-10

**Authors:** Tao Wang, Xin Huang, Li-xue Dai, Kang-min Zhan, Jun Wang

**Affiliations:** ^1^Jiangxi Medical College, Nanchang University, Nanchang, China; ^2^Jiangxi Provincial People's Hospital, The First Affiliated Hospital of Nanchang Medical College, Nanchang, China; ^3^Department of Ophthalmology, Jiangxi Provincial People’s Hospital, The First Affiliated Hospital of Nanchang Medical College, Nanchang, China

**Keywords:** asthma, thalamus, fMRI, functional connectivity, cognitive impairment

## Abstract

**Objective:**

Bronchial Asthma (BA) is a common chronic respiratory disease worldwide. Earlier research has demonstrated abnormal functional connectivity (FC) in multiple cognition-related cortices in asthma patients. The thalamus (Thal) serves as a relay center for transmitting sensory signals, yet the modifications in the thalamic FC among individuals with asthma remain uncertain. This research employed the resting-state functional connectivity (rsFC) approach to explore alterations in thalamic functional connectivity among individuals with BA.

**Patients and methods:**

After excluding participants who did not meet the criteria, this study finally included 31 patients with BA, with a gender distribution of 16 males and 15 females. Subsequently, we recruited 31 healthy control participants (HC) matched for age, gender, and educational background. All participants underwent the Montreal Cognitive Assessment (MoCA) and the Hamilton Depression Rating Scale (HAMD) assessment. Following this, both groups underwent head magnetic resonance imaging scans, and resting-state functional magnetic resonance imaging (rs-fMRI) data was collected. Based on the AAL (Automated Anatomical Labeling) template, the bilateral thalamic regions were used as seed points (ROI) for subsequent rsFC research. Pearson correlation analysis was used to explore the relationship between thalamic functional connectivity and neuropsychological scales in both groups. After controlling for potential confounding factors such as age, gender, intelligence, and emotional level, a two-sample t-test was further used to explore differences in thalamic functional connectivity between the two groups of participants.

**Result:**

Compared to the HC group, the BA group demonstrated heightened functional connectivity (FC) between the left thalamus and the left cerebellar posterior lobe (CPL), left postcentral gyrus (PCG), and right superior frontal gyrus (SFG). Concurrently, there was a decrease in FC with both the Lentiform Nucleus (LN) and the left corpus callosum (CC). Performing FC analysis with the right thalamus as the Region of Interest (ROI) revealed an increase in FC between the right thalamus and the right SFG as well as the left CPL. Conversely, a decrease in FC was observed between the right thalamus and the right LN as well as the left CC.

**Conclusion:**

In our study, we have verified the presence of aberrant FC patterns in the thalamus of BA patients. When compared to HCs, BA patients exhibit aberrant alterations in FC between the thalamus and various brain areas connected to vision, hearing, emotional regulation, cognitive control, somatic sensations, and wakefulness. This provides further confirmation of the substantial role played by the thalamus in the advancement of BA.

## Introduction

Asthma is a condition marked by recurrent wheezing, breathlessness, and coughing. Over 400 million individuals across the globe are affected by asthma. Currently, asthma affects 6.3% of the youth population in China (1). Previous research on the pathological mechanisms of asthma has primarily focused on airway inflammation and airway remodeling (2). However, peripheral inflammatory substances can also impact the central nervous system via various pathways (3). In a prior study, brain MRI scans revealed abnormal changes in the brains of asthma patients (4). Furthermore, markers associated with central nervous degeneration and neuroinflammation have been detected in the blood of asthma patients, suggesting potential consequences of asthma on the central nervous system (5).

The mechanisms through which asthma affects the central nervous system are not yet fully understood. However, it is currently believed that the long-term chronic hypoxia induced by asthma may initiate and exacerbate certain pathophysiological processes, such as reduced perfusion, endothelial dysfunction, and neuroinflammation. In addition to these factors, chemotactic factors produced by airway inflammation can directly traverse the blood–brain barrier, leading to increased release of reactive oxygen species by microglial cells, inducing neuronal apoptosis (6). A recent study found that allergens can reduce communication activity between the amygdala and the respiratory control cortex, exacerbating respiratory difficulties (7). Furthermore, asthma exhibits an overall suppression of the HPA axis, and long-term corticosteroid use also inhibits the HPA axis. This alteration is associated with a reduction in hippocampal volume, thereby impacting working memory (8). Additionally, asthma-related nocturnal sleep disturbances are associated with poorer cognitive function (9). Inherent difficulties in respiratory regulation, cognitive deficits, and emotional control disturbances all contribute to the duration and severity of asthma attacks.

When neuronal metabolic activity is ongoing, local changes in cerebral oxygen content and blood supply occur. fMRI is capable of detecting magnetic field fluctuations resulting from the mismatch between local oxygen consumption and cerebral blood flow in various brain areas, thereby reflecting the metabolic activity of brain areas. Rs-fMRI has increasingly been employed to investigate brain activity alterations in BA patients. Li et al. (10) found abnormal activity in the angular gyrus, prefrontal cortex, temporal gyrus, superior frontal gyrus, and occipital lobe when using fMRI to analyze brain region activity and neural networks in asthma patients. Ritz et al. (11) conducted an fMRI study on individuals with poorly controlled asthma and found increased activation in their dorsal anterior cingulate cortex (dACC). A study revealed abnormalities in these metrics in the frontal and superior lobes of the brains of asthmatic children, closely associated with attention deficits (12). Most of these studies focus on regional activity in the asthmatic brain cortex or changes in connectivity at the network level. In previous research, we found a substantial reduction in bilateral thalamic connectivity in asthma individuals (13). On the other hand, recent research has revealed an inverse relationship between the length of time an individual has had asthma, the level of asthma management, and the frequency of asthma attacks and the volume of the thalamus (14).

The thalamus is often described as an intermediary station for sensory information, playing a pivotal role in not only transmitting sensory input but also participating in cognitive processes. Additionally, it exerts significant control over visceral functions, motor coordination, and the maintenance of cerebral arousal (15). The thalamus acts as a gatekeeper for information directed to the cerebral cortex, selectively enhancing or inhibiting the activation of specific information pathways based on the behavioral state (16). With extensive and global connections to numerous brain regions, the thalamus and its constituent nuclei are likely major hubs within multiple brain networks (17). Consequently, conducting a comprehensive investigation into the functional connections between the thalamus and various other cerebral areas is deemed essential. The rs-FC primarily assesses the statistical associations among signals throughout the entire brain and particular brain areas, demonstrating the potential for exploring how different brain regions coordinate their operations. In this study, we will be the first to employ rs-FC to uncover evidence of abnormal functional connections within the thalamus in asthma patients. We hypothesize that thalamic FC differs from that of the healthy population, and this difference may be implicated in potential neurobiological mechanisms underlying cognitive impairments and emotional dysregulation in asthma patients.

## Materials and methods

### Clinical data

The criteria for selecting asthma subjects are as follows: (1) Adults under the age of 60; (2) presence of recurrent wheezing, with the forced expiratory volume in 1 s (FEV1) falling between 45 and 80% of the predicted normal value; (3) a positive bronchodilator test (FEV1 reversibility of at least 12% and 200 mL after the administration of 200 to 400 μg of salbutamol sulfate); and (4) individuals in a non-acute phase of asthma.

The criteria that are not suitable for the research object are as follows: (1) The presence of other respiratory system diseases; (2) Psychiatric disorders or other chronic diseases that may affect brain structure and function; (3) Drug dependence or adverse habits; (4) Lack of necessary MRI data or clinical assessment information; (5) Maximum head displacement exceeding 2.5 mm in the x, y, and/or z directions, or angle rotation exceeding 2.5 degrees around any axis; (6) Contraindications related to MRI examinations; (7) Absence of claustrophobia and the ability to tolerate MRI examinations. In the end, a total of 31 patients diagnosed with BA participated, comprising 16 males and 15 females. Simultaneously, We selected 31 HCs (16males and 15 females) with basic information matching.

The situations in HCs that are inappropriate for participation are as follows: (1) Presence of asthma or other diseases; (2) Brain or psychological disorders; (3) Completing an MRI examination carries risks; (4) Maximum head displacement exceeding 2.5 mm in the x, y, and/or z directions, or angle rotation exceeding 2.5 degrees around any axis; (5) Incomplete relevant data.

### Neuropsychological assessment

To ensure the reliability of the measured results, we employed the Montreal Cognitive Assessment (MoCA) scale as a cognitive function measurement tool. The maximum score on the scale is 30 points, with an additional point given if the participant has an education level of ≤12 years. Scores between 18 and 26 indicate mild cognitive impairment (MCI) (18). Furthermore, we utilized the 17-item Hamilton Depression Rating Scale (HAMD-17) to evaluate the emotional status of the two groups of participants (19). The test results from this scale will provide a basis for analyzing cognitive and emotional differences among the subjects.

### fMRI data acquisition

Utilizing an 8-channel phased-array head coil, we acquired MRI data employing the Trio 3-Tesla MR scanner from Siemens, Germany. Participants were instructed to remain awake but refrain from engaging in any cognitive activities. Head motion was minimized using a foam cushion, and noise interference was mitigated through the use of earplugs. The parameters for obtaining fMRI images are shown in Table 1.

**Table 1:** Magnetic resonance imaging acquisition parameters.

Parameters	GRE-EPI	T1WI
Matrix size	64 × 64	256 × 256
Field of view	240 × 240 mm	240 × 240 mm
Echo time	40 ms	2.26 ms
Repetition time	2,000 ms	1,900 ms
Slice thickness	4.0 mm	1.0 mm
Slice gap	1 mm	0.5 mm
Flip angle	90°	9°

GRE-EPI, gradient echo sequences echo planar imaging; T1WI, T1-weighted images.

### Data preprocessing

To begin, we validated the quality of the MRI data using the MRIcro software package (Montreal Neurological Institute, Canada) and excluded those with poor quality. Subsequently, we employed the DPABI V5.0 toolbox for brain imaging data processing and analysis to preprocess the fMRI data. This toolbox operates on the MatLab 2018b platform (Mathworks, Natick, MA, United States). We perform data preprocessing, including the following steps: (1) Converted the DICOM (Digital Imaging and Communications in Medicine) format to the NIFTI (Neuroimaging Informatics Technology Initiative) format (2) Discarding the first 10 time records (3) Correction of temporal differences between slices (4) Correction of head motion exceeding 2 millimeters or 2 degrees (5) Alignment and division of functional and structural images, normalized to the standard space, with Resampling to 3 mm x 3 mm x 3 mm Voxel Units (6) An isotropic Gaussian kernel with a Full-Width at Half-Maximum (FWHM) value of 6 mm was applied for spatial smoothing. Temporal filtering in the frequency range of 0.01–0.08 Hz was used to address linear drift (7) The regression covariates included the Friston-24 head motion parameters, as well as white matter, cerebrospinal fluid, and scale scores.

### Functional connectivity analysis based on seed regions

To begin, we adopted a cubic size of 6 × 6 × 6 mm for the bilateral thalamus, designated as our region of interest (ROI). This choice ensured thorough coverage of critical thalamic regions while mitigating biases associated with oversized or undersized ROIs. Next, we established the thalamic ROI in the standardized MNI (Montreal Neurological Institute) space using the AAL (Automated Anatomical Labeling) template. This template, offering detailed anatomical partitioning of the brain, facilitated the creation of a uniform reference framework for consistent cross-individual comparisons. Subsequently, we employed affine transformation and non-linear transformation via SPM (Statistical Parametric Mapping) software to accurately map the ROI to individual subject space. This step was essential for accommodating anatomical variability across individuals and ensuring the precision of functional connectivity analyses. Once the thalamic ROI was accurately defined and mapped, we computed Pearson correlation coefficients between the bilateral thalamus and every voxel in the entire brain. These correlation coefficients underwent Fisher’s Z transformation to approximate a normal distribution. The resulting Z values represented the strength of functional connectivity, offering a quantitative assessment of the functional interactions between the thalamus and the broader brain network.

### Statistical analysis

We conducted statistical analyses using SPSS 27 software, and continuous data were presented as means ± standard deviations. Group comparisons for age, level of education, and BMI were performed using independent sample t-tests. Additionally, a one-way ANOVA was conducted to compare the scores of psychological assessments and cognitive functions, adjusting for the effects of age, gender, and educational level. After controlling for potential confounders such as age, gender, intelligence, and emotional level, a two-sample t-test was further employed to explore the differences in thalamic functional connectivity between the two groups of participants. Subsequently, the DPABI software was employed to analyze intergroup differences while considering age, education level, and head motion as covariates. Utilizing AlphaSim for correction, *p* < 0.05. Finally, the xjView software was utilized to report the locations of brain regions with significant functional connectivity.

## Results

### Demographic statistics and clinical scales

In our investigation, there were no notable disparities in terms of age, gender, body weight, or BMI between the BA patients and HCs (*p* > 0.05). The findings are displayed as the mean value with its corresponding standard deviation (SD). The duration of asthma in our study was observed to be 24.12 years, with a standard deviation of 4.61 years. The HAMD scores for the BA group and the HC group are 8.13 ± 2.39 and 4.74 ± 1.77, respectively, while the MoCA scores for the BA group and the HC group are 25.52 ± 1.93 and 27.55 ± 1.34, respectively. The BA group shows mild cognitive impairment and emotional changes. Please refer to Table 2 for detailed results.

**Table 2:** Demographics and clinical measurements by group.

Conditions	BA	HC	t	*p*-value
Male/female	16/15	16/15	N/A	1
Age (years)	46.23 ± 8.41	47.41 ± 7.30	−0.590	0.557
Education (years)	11.86 ± 1.52	12.47 ± 1.31	−1.693	0.096
BMI (kg/m^2^)	21.64 ± 2.53	21.88 ± 2.74	−0.358	0.721
Duration of asthma	24.12 ± 4.61	N/A	N/A	N/A
HAMD score	8.13 ± 2.39	4.74 ± 1.77	6.340	<0.001
MoCA score	25.52 ± 1.93	27.55 ± 1.34	−4.819	<0.001

BA group, bronchial asthma group; HC group, healthy control group; BMI, body mass index; HAMD, Hamilton Depression Scale; MoCA, Montreal Cognitive Assessment; *p* > 0.05.

### Differences in resting-state functional connectivity between the two groups

**Figure 1:**
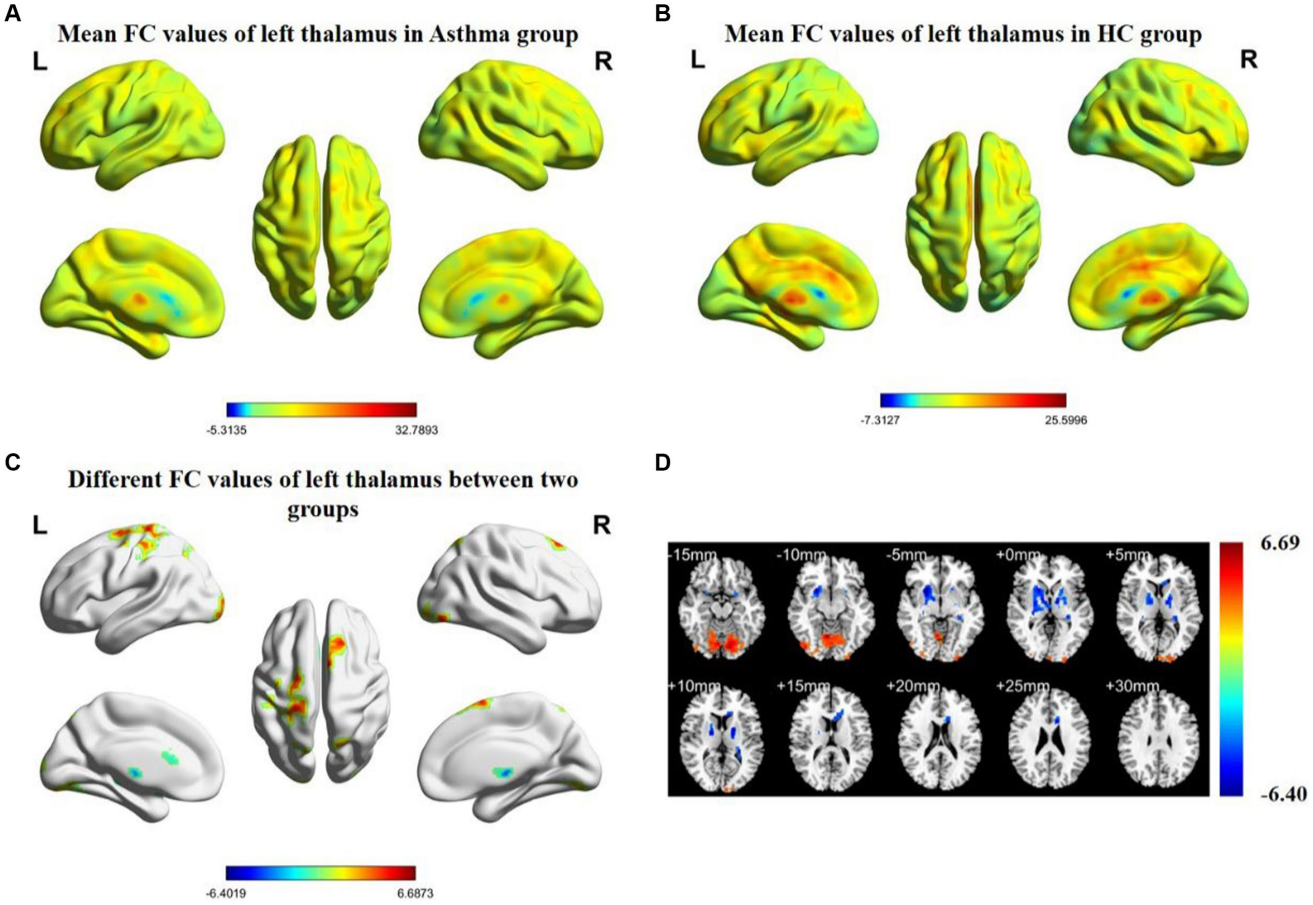
Spatial distribution maps of functional connectivity (FC) in the brain regions of the bronchial asthma group **(A)** and the healthy control group **(B)**, based on the seed point of the left thalamus. **(C, D)** The brain regions where there are significant differences in FC between the two groups (with a voxel-level *p*-value less than 0.05, corrected using AlphaSim). Color bars indicate t-scores; cool colors indicate regions in BA with lower FC values compared to HC, while warm colors indicate the opposite.

**Figure 2:**
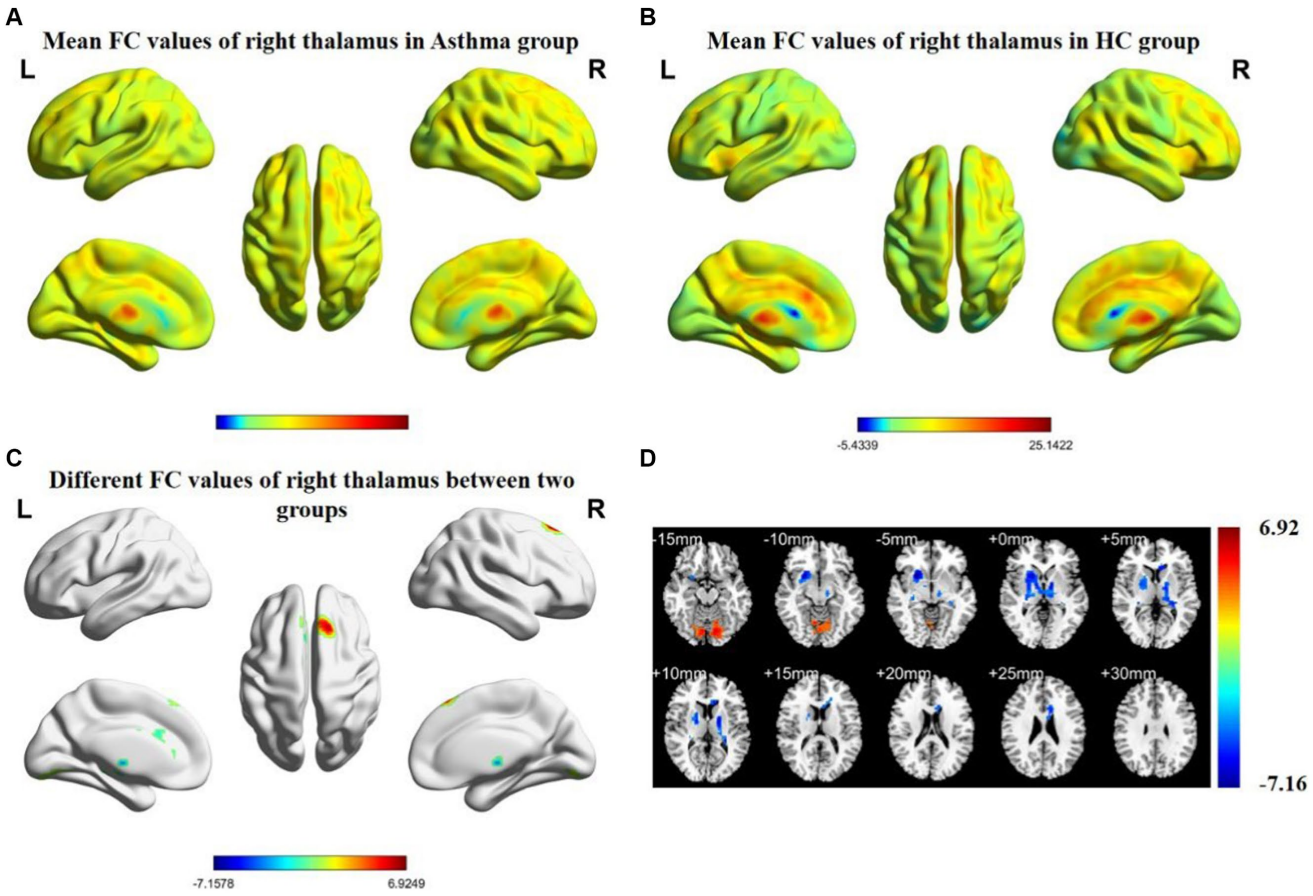
Spatial distribution maps of functional connectivity (FC) in the brain regions of the bronchial asthma group **(A)** and the healthy control group **(B)**, based on the seed point of the right thalamus. **(C, D)** The brain regions where there are significant differences in FC between the two groups (with a voxel-level *p*-value less than 0.05, corrected using AlphaSim). Color bars indicate t-scores; cool colors indicate areas in BA with lower FC values compared to HC, while warm colors indicate the opposite.

The results of inter-group rs-FC comparisons based on bilateral thalamic seed regions and voxels from other brain areas are presented in Table 3. In comparison to the HC group, the BA group exhibited increased FC between the left thalamus and the left cerebellar posterior lobe (CPL), left postcentral gyrus (PCG), and right superior frontal gyrus (SFG). Simultaneously, it demonstrated decreased FC with both the lentiform nucleus (LN) and the left corpus callosum (CC). The right thalamus showed increased FC with the right SFG and the left CPL, while FC with the right LN and the left CC decreased. The anatomical locations, voxel sizes, and corresponding MNI peak coordinates of differential brain regions between the two groups are detailed in Table 3. Figures 1, 2 depict the brain regions where notable alterations in bilateral thalamic rs-FC were observed in both groups.

**Table 3:** Differences in FC with the thalamus were observed in brain regions between two groups.

Seed-ROIs	L/R	Brain area	Voxel	MNI			t-value
				X	Y	Z	
**Left thalamus**							
	L	CPL	205	−12	−81	−18	6.6873
	L	LN	91	−12	−3	0	−6.1981
	R	LN	158	24	12	−6	−6.4019
	L	CC	59	−12	15	24	−4.9463
	L	PCG	81	−24	−33	72	6.0385
	R	SFG	102	6	6	69	5.6002
**Right thalamus**							
	L	CPL	82	−15	−78	−18	5.7834
	R	LN	214	21	18	−6	−7.1578
	L	CC	62	−9	27	6	−5.8531
	R	SFG	95	18	27	60	6.9249

Voxel level *P* < 0.05, AlphaSim corrected. MNI, Montreal Neurological Institute; CPL, Cerebellum Posterior Lobe; LN, Lentiform Nucleus; CC, Corpus Callosum; PCG, Postcentral Gyrus; The negative *t*-values are decreased functional connectivity in BA vs. control, and that the positive *t*-values are increased functional connectivity.

### Correlation analysis

**Figure 3:**
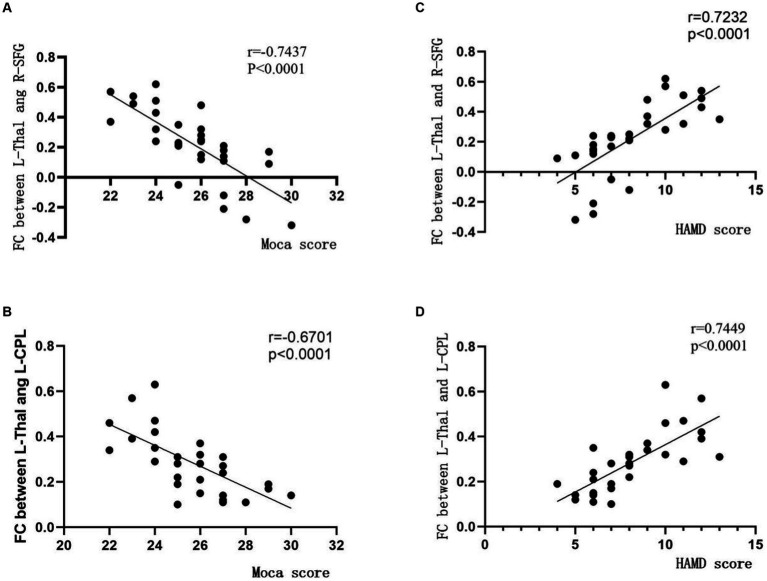
Correlation between inter-group differential brain regions based on thalamus functional connectivity analysis and scores on neuro-psychological assessment scales. The vertical axis represents the functional connectivity (FC) values, while the horizontal axis corresponds to the scores on the neuro-psychological assessment scales. MoCA, Montreal Cognitive Assessment; HAMD, Hamilton Depression; Thal, thalamus; SFG, superior frontal gyrus; CPL, cerebellar posterior lobe; L for Left; R for Right.

Partial correlation analysis revealed a negative correlation between the FC values of the left thalamus (L-Thal) and right superior frontal gyrus (R-SFG) in the BA group and MoCA scores (r = −0.7437, *p* < 0.0001, Figure 3A), as well as a positive correlation with HAMD scores (r = 0.7232, *p* < 0.0001, Figure 3C). Furthermore, the FC values of L-Thal and left cerebellar posterior lobe (L-CPL) showed a negative correlation with MoCA scores (r = −0.6701, *p* < 0.0001, Figure 3B) and a positive correlation with HAMD scores (r = 0.7449, *p* < 0.0001, Figure 3D). The FC values between the bilateral thalamus and other differential brain regions did not show significant correlations with neuro-psychological measurement assessments.

## Discussion

Previous research has found a significant decrease in voxel-mirrored homotopic connectivity (VMHC) between the bilateral thalamus in asthma patients, reflecting specific changes in internal information exchange, integration, and coordination within the thalamus (13). However, unlike discussions on functional changes between homologous regions of the bilateral thalamus with VMHC, functional connectivity (FC) analysis focuses more on alterations in the interaction between the thalamus as a key relay station and different brain regions (20, 21). This study is the first to look into rs-FC in BA patients between the thalamus and other brain areas. The principal discoveries of this study demonstrate that individuals with asthma show extensive FC deficiencies in multiple brain regions associated with emotion regulation, cognitive control, somatic sensation, arousal, visual processing, and auditory processing. These findings align with our initial hypothesis. Despite statistical analyses revealing only mild cognitive impairment and emotional abnormalities in the BA group based on the scores from the two assessment scales, subsequent correlation analyses unveiled potential neurobiological indicators reflecting cognition and emotion. Specifically, the functional connectivity strength between the left thalamus (L-Thal) and the right superior frontal gyrus (R-SFG), as well as the left cerebellar posterior lobe (L-CPL), was identified.

The cerebellum posterior lobe (CPL) constitutes a significant portion of the human cerebellum (22). The thalamus conveys signals originating from the dentate nucleus of the CPL and subsequently projects them to the cerebral cortex, providing processed information back to the cerebellum (23). This neural circuit is typically associated with precision task execution, visual processing, speech expression, and emotional responses (24, 25). Similarly, we observed a significant correlation between increased functional connectivity (FC) between the thalamus and cerebellar posterior lobe (CPL) and the presence of cognitive impairment and emotional abnormalities. Aberrant FC results in a decline in functional interaction capacity between brain regions, thereby weakening the efficiency of information transmission. In research examining primary nocturnal enuresis in children, disturbances in arousal were linked to diminished functional connectivity between the thalamus and CPL (26). In the study conducted by Liu and colleagues (27), it was also observed that individuals suffering from sleep disorders exhibited elevated metabolic activity in both the thalamus and the CPL. Xie et al. (28) investigated the spontaneous brain activity in patients with obstructive sleep apnea (OSA) using the percentage of amplitude fluctuation (PerAF). Their findings revealed an increased PerAF in the CPL, which correlates with the presence of sleep disturbances. Another study also indicates enhanced regional activity in the left CPL in BA patients (29). In our study, we observed enhanced FC between the Thal and CPL. This may potentially amplify projections to the cortical areas of CPL, resulting in cortical excitation. This could possibly explain the higher prevalence of nocturnal awakenings and sleep disturbances in asthma patients. Prolonged insufficient sleep can lead to decreased vigilance, resulting in reduced responsiveness of individuals with asthma to external threats (30, 31). This may contribute to an increased risk of triggering asthma-related behaviors. Research indicates that, compared to non-depressed individuals with asthma, those with depression exhibit significantly increased cerebral blood flow in the CPL (32–34). The inclination toward depression is also related to CPL metabolism (35). Furthermore, as a region with a “proofreading” function for information, the increased signal strength in the cerebellum reflects abnormal control of respiratory muscles, and this change in asthmatic individuals may be associated with alterations in their respiratory rate (36, 37). It is worth noting that in the cerebellum-thalamus-cerebral cortex (CTC) ascending loop, we have only identified FC changes between the cerebellum and thalamus in BA patients. In the future, probabilistic fiber tractography can be used to determine the regions with strong fiber bundle connections between the thalamus and the cerebellum, serving as ROIs for the projection of the cerebellum onto the thalamus. This approach can be employed to construct the FC between the thalamus and the cortex within this circuit, thereby elucidating the specific changes within the CTC in individuals with asthma.

LN, including the pallidum and the putamen nucleus, is a critical part of the basal ganglia, projecting to the ventrolateral nucleus of the thalamus and the lateral geniculate body (38). The basal ganglia-thalamus-cortical loop (BTC) is involved in arousal and attention functions (39). It also mediates regulatory control over a wide range of cortical areas, playing a pivotal role in emotional processing, motor control, cognitive processing, and motivational behavior (40). In our study, we observed a reduction in FC between the left thalamus and bilateral LN, while the right thalamus exhibited decreased FC, specifically with the right LN. The altered FC patterns may contribute to an overall decrease in information transmission efficiency between the thalamus and the cerebral cortex. Previous fMRI studies have indicated that the pallidum and the putamen nucleus often exhibit abnormal spontaneous activity when individuals are experiencing emotions such as fear, anxiety, and sadness (41). Individuals with asthma are more likely to trigger anxious thoughts about the dire consequences of an asthma attack. Additionally, it is widely hypothesized that the aberrant neural activity in this circuit serves as a fundamental neural mechanism in individuals with obsessive-compulsive disorder (42). Decreased thalamic gating efficiency can lead to a greater projection of intrusive, distressing thoughts into the cortex (16). Under the influence of obsessive thoughts and anxious emotions, this can result in more frequent oral corticosteroid and bronchodilator use. Prolonged corticosteroid treatment is linked to a decrease in LN volume, which in turn leads to more severe anxiety occurrences (14, 43). Furthermore, damage to dopaminergic neurons in LN is closely associated with cognitive impairments in schizophrenia, including deficits in attention, working memory, reward processing, and executive functions (44). The LN is involved in the reward processing system and is responsible for linking different sensory cues with rewarding outcomes. In individuals suffering from severe depression, LN becomes abnormally activated during the occurrence of negative emotions, thereby intensifying the avoidance motivation to alleviate the experience of negative emotions (45, 46). Impairments in reward and executive functions in BA patients may lead to reduced proactiveness in seeking medication and a higher likelihood of experiencing negative emotions. In the future, it is also possible to integrate multimodal data from DTI and fMRI to segmentally construct a detailed profile of BTC changes in BA patients.

The SFG is situated in the anterior medial prefrontal cortex, a key region within the default mode network (DMN), and is primarily involved in functions such as attention selection, inhibitory control, stress perception, and working memory (47, 48). The DMN consumes a substantial amount of energy during rest, making it particularly susceptible to oxidative stress damage and the influence of diseases (49). Previous research has shown reduced activity in other brain regions of the DMN in individuals with asthma, such as the angular gyrus and the precuneus, confirming regional functional and network-level intrinsic activity abnormalities in the brains of BA patients (10). Hwang et al. (17) discovered robust functional connections between the DMN and various thalamic nuclei. Therefore, we hypothesize that, to compensate for the reduction in internal network activity, the thalamus and SFG’s FC will increase adaptively. The thalamus mediates top-down regulation and the filtering and integration of sensory information, as well as serving as a crucial hub for selective inhibition of external stimuli and focused attention (50). Meanwhile, the prefrontal cortex plays a central role in processing higher-level emotional and cognitive information. The enhanced functional connection between SFG and the thalamus is considered a neurofunctional characteristic of schizophrenia, often characterized by excessive attention to external stimuli and abnormal emotional reactions to these stimuli (51). Furthermore, these changes in BA patients may lead to a reduction in the use of adaptive strategy, potentially resulting in emotional regulation disorders (52, 53). Research indicates a positive correlation between spontaneous activity in SFG and perceived stress, with excessive perceived stress typically stemming from negative emotions such as anxiety and depression (54). The thalamus also plays a role in mediating noxious input to the cortex, and the functional reorganization of the SFG is crucial in top-down modulation of pain experiences, with strong FC between them potentially contributing to the sustained perception of pain (55). The thalamus and SFG’s FC serve as a shared pathway in the experiences of breathlessness and pain perception (56, 57). We hypothesize that asthma patients, in the long-term management of their breathing difficulties, gradually adapt to the stress associated with pain, transforming their adaptation to breathlessness into a tolerance for pain. This adaptation is manifested through increased spontaneous activity in the superior frontal gyrus (SFG). Therefore, the heightened functional connectivity (FC) between the thalamus and SFG in BA patients may be linked to emotional regulation disorders, perceived stress, and experiences related to pain and breathlessness.

PCG is a crucial region within the sensorimotor network responsible for processing various types of sensory perceptions. Sensory stimuli are conveyed through C-fibers to the spinal thalamic tract and are then relayed to the PCG via the thalamic ventral posterior nucleus (58). In their analysis of gender differences in chronic cough, Morice et al. (59) found that females exhibited higher sensitivity in their cough reflex, which was associated with increased activation in the PCG. Our research has revealed that, in comparison to HCs, asthma patients exhibit increased FC between the thalamus and PCG. This finding aligns with the tendency for asthma patients to experience coughing more frequently. Furthermore, damage to the PCG cortex could potentially affect respiratory motor control. Zhang et al. (60), in their fMRI analysis of COPD patients, observed enhanced low-frequency amplitude of fluctuations (ALFF) in the PCG, which was closely related to over-breathing. As the condition worsens, patients with asthma may gradually experience decreased sensitivity in perceiving asthma symptoms within the somatosensory cortex. This decline in perception sensitivity may hinder their ability to promptly address their condition due to reduced perception strength (61). Previous research has yielded varying results, showing reduced functional connectivity (FC) between the insular cortex and the PCG in BA patients (42). This reduction may be attributed to pathological activation in the PCG, which subsequently diminishes the FC between the insular cortex and PCG. Furthermore, early cognitive development often relies on essential sensory experiences, and excessive input from the thalamus can lead to aberrant sensory experiences, ultimately impacting cognitive function (58). Hence, the PCG is involved in the occurrence of bronchial hyperreactivity and respiratory overdrive in asthma, and it can serve as a potential biomarker for the severity of asthma symptoms.

Lastly, we have observed a decrease in FC between the CC and the thalamus, even though there is no apparent anatomical correlation between them. But functionally, they are interconnected, such as in the context of visual short-term memory capacity (62), visual processing (63), and speech formation (64). In fact, the input from the thalamus plays a highly instructive role in the maturation trend of the CC area, achieved through the regulation of neuronal projection, development, signal transduction, and activity-dependent plasticity (65, 66). The CC serves as an intermediary for interhemispheric communication (67) and indirectly contributes to cognitive functions like language, attention, working memory, and visual spatial memory (68–70). Previous studies have suggested that CC atrophy in BA patients might be mediated by allergens (71) and affect the cortical metabolism associated with cognitive functions (72). In BA patients, there is a reduction in the FC between the CC and the thalamus, which we hypothesize is associated with a decline in the coordinated processing of sensory stimuli across both brain hemispheres. This decline includes the ability to recognize odors from olfactory stimuli (73, 74). This might explain why asthma patients have difficulty identifying harmful gases, which can trigger asthma symptoms. In an fMRI study, Wang et al. (75) found that damage to the white matter integrity of the CC is related to the severity of anxiety. Changes in FC between the thalamus and these brain structures contribute to understanding the pathophysiological mechanisms underlying cognitive impairments, emotional dysregulation, and motor control failures in BA patients. In the future, inducing positive plastic changes may be considered in the treatment of BA to promote brain function recovery.

## Limitations

Nonetheless, there are certain constraints associated with this study. In the first place, the sample size was rather limited owing to rigorous inclusion criteria. Furthermore, during the administration of the neuro-psychological assessment scales, it is noteworthy that the subjects may not have fully adhered to a conservative representation of their condition, introducing a potential bias to the test results. Thirdly, this study did not investigate how these FC differences change over time. Future research could employ dynamic FC analysis to elucidate this aspect. Hence, it is imperative to conduct longitudinal studies using a variety of analytical approaches for fMRI. This will enable us to investigate the evolving patterns in FC as time progresses. Fourthly, our study only identified brain regions where there were differences in FC between the BA group and the HCs concerning the thalamus and other cortical regions. In the future, machine learning methods such as support vector machine (SVM) classifiers can be employed to determine whether these brain regions can serve as discriminative features for distinguishing these groups.

## Conclusion

In our study, we have verified the presence of aberrant FC patterns in the thalamus of BA patients. When compared to HCs, BA patients exhibit abnormal changes in functional connectivity between the thalamus and various brain regions associated with vision, hearing, emotional regulation, cognitive control, somatic sensations, and wakefulness. This provides further confirmation of the substantial role played by the thalamus in the progression of BA.

## Data availability statement

The original contributions presented in the study are included in the article/supplementary material, further inquiries can be directed to the corresponding author.

## Ethics statement

The studies involving humans were approved by Ethics Committee of Jiangxi Provincial People’s Hospital. The studies were conducted in accordance with the local legislation and institutional requirements. The participants provided their written informed consent in this study.

## Author contributions

TW: Conceptualization, Data curation, Formal analysis, Investigation, Methodology, Software, Writing – original draft. JW: Funding acquisition, Supervision, Writing – review & editing. XH: Methodology, Resources, Software, Writing – review & editing. L-xD: Investigation, Methodology, Writing – review & editing. K-mZ: Investigation, Methodology, Writing – review & editing.
